# Development of an outcome prediction tool for patients considering a total knee replacement – the Knee Outcome Prediction Study (KOPS)

**DOI:** 10.1186/1471-2474-15-451

**Published:** 2014-12-23

**Authors:** Tim Barlow, Mark Dunbar, Andrew Sprowson, Nick Parsons, Damian Griffin

**Affiliations:** Warwick Medical School, Clinical Sciences Research Laboratories, University Hospitals of Coventry and Warwickshire, Clifford Bridge Road, Coventry, CV2 2DX UK

**Keywords:** Total knee replacement, Patient factors, Outcome prediction tool

## Abstract

**Background:**

Knee osteoarthritis affects 10% of the UK population over 55 years, resulting in pain and decreased quality of life. Knee replacement surgery has a proven benefit, with over 85,000 performed each year in the UK; however, approximately 17% of people are dissatisfied after surgery. Consequently, some Primary Care Trusts have reduced the funding available for knee replacements.

Most previous work has focused on the effect of different prostheses and treatment protocols on patient’s outcome. However, this has been unable to account for all the variability and there is growing evidence that patient factors may significantly affect outcome. How to identify these at risk patients has been identified as a research priority by the National Institute of Clinical Excellence, the British Orthopedic Association, and the National Joint Registry.

The aim of this study is to develop a clinically appropriate outcome prediction tool based on measurable predictors affecting outcome.

**Methods/design:**

We propose a prospective cohort study, designed to develop and validate an outcome prediction tool based on patient factors.

Six hundred patients who are scheduled for total knee replacement secondary to primary osteoarthritis will be recruited before surgery from all six hospitals (NHS and private) that provide total knee replacements to the population of Coventry and Warwickshire (UK). Patients will complete a baseline assessment of patient factors before their operation and will be followed up at 6 and 12 months post surgery.

**Discussion:**

A clinically appropriate outcome prediction tool will allow patients to make a more informed decision regarding surgery. Aligning patient expectations with a realistic prediction of outcome should improve satisfaction. Ultimately, this project is likely to inform national policy making and regional service provision.

## Background

Primary osteoarthritis (OA) of the knee is a condition that can lead to loss of knee function. This in turn can lead to difficulty working, performing activities of daily living, stress, and depression [1]. Ten percent of the U.K. population over the age of 55 suffers from pain as a result of knee OA [2]. With an ageing population, this condition will present an ever increasing health burden. Surgery in the form of total knee replacement has reliably been shown to have a beneficial effect [3], and 95,454 knee replacements were performed in England and Wales in 2013, with over 90% of these for OA [4]. However a sub group of patients exist who have poor outcomes following knee replacement. Some studies show chronic pain rates as high as 17% [5]. Most previous work has focused on the effect of different prostheses and treatment protocols on outcome. However these factors have proved insufficient to account for all the variability in the outcome, and there is growing evidence that patient factors may significantly affect outcome [6]. A patient factor can be defined as being any factor that is intrinsic to the patient and that is not rapidly changed by a change in environment. These factors may include demographic data, functional and general health scores as well as psychological attributes and radiographic appearances.

Several patient factors have been identified as accounting for some of the variance in outcome after knee replacement; however which patient factors are important, and how much they affect outcome remains uncertain.

Pre-operative functional scores have been shown in several studies to have a consistent correlation to post operative function scores [7–10]. This would suggest that patients who are operated on earlier in their clinical course have a better outcome.

Medical co-morbidity has also been shown to have an effect on postoperative function [5, 9, 11–13], although some studies of reasonable size have not found a correlation [6, 7, 10, 14].

Psychosocial factors have been examined by several authors. Sharma [10] included the role function, emotional, social function and motivation subscales of the SF-36 in their hierarchical regression model, where an additional 19% of the variance of three-month post-op SF-36 PF was explained. Heck [7] and Lingard [9] used slightly different measures of mental health but both showed a significant association with postoperative physical function.

There is some evidence to suggest that an increased body mass index (BMI) is correlated with a worse absolute physical outcome. However, Spicer [14] noticed that there was no significant difference in the absolute improvement in physical outcome. Fortin [8] reported information on the change scores for preoperatively determined high function and low function groups. The lower function groups, although having lower absolute six month function scores, showed greater improvement than the high functioning group.

Age, gender and education had either non-significant results, or produced correlations that were too small to be of clinical significance in the above papers. However, it may be that these patient factors were, at least in part, confounded with one or other of the factors that showed positive associations with outcome.

Stratification of patient risk is currently the most important question in knee replacement surgery, and has been identified as a research priority by the U.K. National Institute of Health and Care Excellence [13].

Previous pilot work at out institution has identified measurable pre-operative patient factors that may affect outcome. The pilot work has allowed the development of a protocol that will enable measurement of the effect of different patient factors.

The primary aim of this study is to measure the effect of patient factors on outcome after knee replacementand to develop an outcome prediction tool for patients considering a total knee replacement. This tool could be used to empower patients to make a more informed decision about having a knee replacement, by allowing an accurate, personalised prediction of what they can expect.

## Methods/design

We plan to undertake a prospective multi-centre cohort study including all six hospitals (NHS and private) that provide total knee replacements to the population of Coventry and Warwickshire. This represents a diverse population and basic demographic data will be compared to data from the National Joint Registry (NJR) [4] to allow comparison between the study population and the U.K. as a whole.

This study will use methodologies tested in a pilot study undertaken at our institution. This study tested the feasibility of the recruitment procedure and the type and presentation of questionnaires used to measure patient factors.

A consecutive series of patients presenting at clinics at each centre will be approached and if deemed eligible will be asked to consent to take part in the study. Our pilot study showed that this procedure is feasible and that 80% of eligible patients give consent to participate. Based on this figure, we expect to recruit about 60 patients per month, over a recruitment period from April 2013 to July 2014. This should provide a pool of over 1000 patients who are eligible for inclusion in the study, and over 800 who would consent to participate. We have set our recruitment target to be somewhat lower than this to allow for any unexpected recruitment problems. We do not anticipate any serious problems in reaching this target; however, the pilot study took place at one institution, and there may be a drop in recruitment rate when extending the study over multiple sites.

Patients will be recruited from their pre-operative clinic appointment where a baseline assessment of patient factors will take place. Factors assessed include: age; BMI; social support (measured by living alone); deprivation (postcode); knee status and knee pain (Oxford Knee Score - OKS); general health (Short Form-36 - SF-36, which has eight domains across two subscales (mental and physical functioning)); medical co-morbidity (Co-morbidity questionnaire); joint co-morbidity (Joint Co-morbidity questionnaire); psychological co-morbidity (Hospital Depression and Anxiety Score); helplessness and coping style (Arthritis Helplessness Index); expectation (Knee Expectation Questionnaire); and radiographic status (Ahlback Score).

Patients will then be followed up at six and twelve months by postal questionnaire using the OKS, the SF-36, and a satisfaction score.

### Setting

Patients listed for a knee replacement within the entire Arden Primary Care Cluster (private and NHS hospitals) will be screened for eligibility.

The participating hospitals are: University Hospital Coventry (University Hospitals of Coventry and Warwickshire NHS Trust (UHCW)); Hospital of St. Cross, Rugby (UHCW); Warwick Hospital (South Warwickshire Hospital NHS Trust); George Elliot Hospital (George Eliot Hospital NHS Trust); BMI Meriden Hospital, Coventry; and Nuffield Hospital Warwick, Leamington Spa.

### Eligibility criteria

Patient population inclusion criteria:

- Patients who have a diagnosis of primary osteoarthritis
- Managed with a primary total knee replacement during the study period
- Able to complete questionnaires and give informed consent
- Patients who are over 50 years

Patient population exclusion criteria:

- Those who are unable or unwilling to give informed consent
- Those whose knee replacement is a unicompartmental or a revision procedure

### Outcome measures

The primary outcome measure is knee function as measured by the OKS [15] at one year after operation. This condition-specific measure is a 12-item Patient Reported Outcome Measure (PROM), specifically designed to test knee pain and function. It has been well validated for this group of patients and is used by the U.K. Department of Health in the National Joint Registry.

The secondary outcome measures are the SF-36 [16], a 36 item patient reported measure of general health, which measures eight domains of health, including both physical and mental wellbeing, and a satisfaction score (validated in the pilot work).

Additional data about the process of inpatient treatment will be collected to ensure that there are no important differences in the treatment experiences between patients (e.g. surgical technique or length of stay).

#### Sample size

We have designed this study to have an 80% power to detect associations, at the 5% level, between pre-operative factors and outcome, with a correlation coefficient of 0.2. This will predict factors that account for more than 4% of the variation in the primary outcome measure, which is below the clinically detectable changes for the OKS. To do this we need to recruit 400 patients (calculation using the pwr package in R, which implements the methods of Cohen (1988) for a linear model) [17].

We plan to use cross-validation methods for model development. As this will effectively reduce the sample of data we use for model fitting by a small factor, we choose to increase the sample size by 20%. Therefore, we require 480 patients with complete follow-up data.

Similar cohort studies have demonstrated a loss to follow up of around 10-15% [5–10]. We expect a loss to follow-up of about 10-15%, but have allowed for 20%. Therefore, our cohort would have to recruit 600 participants to be able to ensure analysis of 480 patients.

### Data analysis

The primary analysis will use multiple linear regression models to identify patient factors that are significantly associated with the OKS and the SF-36 (the primary and secondary outcome measures – both have been validated for this group of patients). Logistic regression models to assess dichotomous outcomes (satisfaction) will also be used. Other factors that may affect outcome (e.g. level of experience of surgeon) will also be incorporated into the model. Diagnostic analysis will be used to assess model assumptions. Cross-validation techniques (e.g. 10 fold cross validation) will be used to inform model building and predictive power [18].

In our study that validated the tools we are using, 2.4% of cases had missing data at baseline assessment. Patterns of missingness will be investigated, for instance using missingness as the response variable in a logistic regression model, to assess whether there is any non-random element to the missing data.

We expect a loss to follow up of 10-15% and will use complete case analysis of follow up data as the primary analysis. As a sensitivity analysis to explore the effects of missing data we will use multiple imputation using standardised methods available through statistical packages (e.g. Multiple Imputation using Chained Equations). Results of both imputational analysis and complete case analysis will be presented.

Factors that predict outcome will form part of a streamlined questionnaire. The statistical analysis will allow weighting of the included factors, providing an estimate of outcome. Therefore, the outcome prediction tool will consist of both a streamlined questionnaire, and an associated algorithm.

High-level analysis will be undertaken in R [19] and also some data management and validation in SPSS (IBM SPSS Statistics for Macintosh, Version 22.0.), under the direction of an experienced statistician (NP).

### Regulatory approval

This study has been approved by the Northampton National Research Ethics Service (12/EM/0336), and all relevant local approvals at all participating sites.

## Discussion

This paper describes the justification for conducting a multi-centre cohort study using patient factors to predict outcome in patients after knee replacement surgery. By measuring the patient factors that are associated with better or worse functional outcome, we plan to predict outcome in individual patients, and support clinical decision making, hence improving quality of care. It will also facilitate interpretation of evidence from published observational studies of different interventions by determining whether groups of patients were similar, and allow post-hoc adjustment of clinical effectiveness studies for risk profile. The ability to adjust samples for case mix is particularly topical in view of the recent National Joint Registry introduction of Patient Reported Outcome Measures for total knee replacement. This data is in line with Department of Health recommendations and is used in service allocation.

The main strength of this study is the breadth and comprehensiveness of the patient factors selected for inclusion in the model building process; many of these factors have previously been shown to correlate with important outcome after surgery. Another strength is the size and demographic of the patient population sampled, which includes both NHS and private patients.

## Abbreviations

OA: Primary osteoarthritis
BMI: Body mass index
OKS: Oxford Knee Score
SF-36: General health Short Form questionnaire.

## References

[1] Smith BW, Zautra AJ (2008). The effects of anxiety and depression on weekly pain in women with arthritis. Pain.

[2] Peat G, Thomas E, Duncan R, Wood L, Hay E, Croft P (2006). Clinical classification criteria for knee osteoarthritis :performance in the general population and primary care. Ann Rheum Dis.

[3] Juni P, Reichenbach S, Dieppe P (2006). Osteoarthritis: Rational approach to treating the individual. Best Pract Res clin Rheumatiol.

[4] National Joint Registry for England and Wales. 2014,http://www.njrcentre.org.uk/njrcentre/Reports,PublicationsandMinutes/Annualreports/tabid/86/Default.aspx,

[5] Hawker G, Wright J, Coyte P, Paul J, Dittus R, Croxford R, Katz B, Bombardier C, Heck D, Freund D (1998). Health-related quality of life after knee replacement. J Bone and Joint Surg Am.

[6] Santaguida PL, Hawker GA, Hudak PL, Glazier R, Mahomed NN, Kreder HJ, Coyte PC, Wright JG (2008). Patient characteristics affecting the prognosis of total hip and knee joint arthroplasty: a systematic review. Can J Surg.

[7] Heck DA, Robinson RL, Partridge CM, Lubitz RM, Freund DA (1998). Patient outcomes after knee replacement. Clin Orthop.

[8] Fortin PR, Clarke AE, Joseph L, Liang MH, Tanzer M, Ferland D, Phillips C, Partridge AJ, Bélisle P, Fossel AH, Mahomed N, Sledge CB, Katz JN (1999). Outcomes of total hip and knee replacement: preoperative functional status predicts outcomes at six months after surgery. Arthritis Rheum.

[9] Lingard EA, Katz JN, Wright EA, Sledge CB, Kinemax Outcomes Group (2004). Predicting the outcome of total knee arthroplasty. J Bone Joint Surg Am.

[10] Sharma L, Sinacore J, Daugherty C, Kuesis DT, Stulberg SD, Lewis M, Baumann G, Chang RW (1996). Prognostic factors for functional outcome of total knee replacement: a prospective study. J Gerontol A Biol Schi Med Sci.

[11] König A, Scheidler M, Rader C, Eulert J (1997). The need for a dual rating system in total knee arthroplasty. Clin Orthop Relat Res.

[12] Wasielewski RC, Weed H, Prezioso C, Nicholson C, Puri RD (1998). Patient comorbidity relationship to outcomes of total knee arthroplasty. Clin Orthop.

[13] National Institute for Health and Care Excellence: Osteoarthritis: care and management in adults. Available at http://www.nice.org.uk/guidance/cg177 25340227

[14] Spicer DD, Pomeroy DL, Badenhausen WE, Schaper LA, Curry JI, Suthers KE, Smith MW (2001). Body mass index as a predictor of outcome in total knee replacement. Int Orthop.

[15] Murray DW, Fitzpatrick R, Rogers K, Pandit H, Carr AJ, Dawson J (2007). The use of the Oxford hip and knee scores. J Bone Joint Surg Br.

[16] Garratt AM, Ruta DA, Abdalla MI, Buckingham JK, Russell IT (1993). The SF36 health survey questionnaire: an outcome measure suitable for routine use within the NHS?. BMJ.

[17] Cohen J (1988). Statistical power analysis for the behavioral sciences.

[18] Stone M (1974). Cross-validatory choice and assessment of statistical predictions. J R Stat Soc B.

[19] R Core Team. R (2014). A language and environment for statistical computing.

[CR20] The pre-publication history for this paper can be accessed here:http://www.biomedcentral.com/1471-2474/15/451/prepub

